# The lactate–lactylation axis in tumor radioresistance: metabolic, epigenetic, and immune mechanisms with emerging links to RNA regulation

**DOI:** 10.3389/fimmu.2026.1886398

**Published:** 2026-06-24

**Authors:** Yuxiang Zhang, Jiaqi Zhang, Yun Cao

**Affiliations:** 1 Department of Digestive Endoscopy, The First Affiliated Hospital of Dalian Medical University, Dalian, Liaoning, China; 2 Department of Pathology, School of Basic Medical Sciences, Xuzhou Medical University, Xuzhou, Jiangsu, China

**Keywords:** DNA repair, lactate, lactylation, metabolic reprogramming, radioresistance, RNA processing

## Abstract

Radiotherapy remains a cornerstone treatment for solid tumors, but its efficacy is frequently limited by intrinsic and acquired radioresistance. Increasing evidence indicates that lactate metabolism and protein lactylation are not merely by-products of glycolysis, but context-dependent regulators of tumor adaptation to irradiation. In irradiated tumor models, lactate-associated metabolic rewiring has been linked to DNA damage repair, redox buffering, and clonogenic survival. Other mechanisms, including chromatin remodeling, immune suppression, and RNA-level regulation, should be interpreted according to their evidence strength, ranging from direct radiotherapy evidence to mechanistic inference from related cancer or immune models. Beyond its metabolic functions, lactate provides a biochemical context for lysine lactylation, an emerging post-translational and epigenetic modification that may regulate chromatin accessibility, stress-responsive transcription, selected DNA damage response proteins, and immune remodeling. Current evidence also suggests possible intersections with post-transcriptional regulation, including m6A-dependent RNA stability and RNA-binding protein activity. However, these RNA-processing-related mechanisms remain insufficiently validated in radiotherapy models and are discussed here primarily as an emerging, hypothesis-generating layer rather than as an established driver of radioresistance. This review summarizes current evidence linking the lactate-lactylation axis to tumor radioresistance, with emphasis on metabolic adaptation, DNA damage repair, and immunosuppressive remodeling of the tumor microenvironment. We also discuss therapeutic strategies targeting this axis, including MCT and LDH inhibitors, oxidative phosphorylation blockade, indirect modulation of lactylation-associated machinery, and lactate-depleting nanoplatforms. Finally, we highlight unresolved mechanistic questions and future directions for integrating metabolic, epigenetic, immune, and cautiously framed RNA-processing approaches to improve radiosensitization.

## 1 Introduction

Radiotherapy is a primary treatment option for solid tumors, with about half of all cancer patients receiving it during their disease (1, 2). However, its effectiveness is often limited by tumor radioresistance, which leads to local recurrence and poor clinical outcomes (3, 4). Recently, metabolic reprogramming has become recognized as a hallmark of cancer, leading to increased focus on how altered tumor metabolism contributes to therapy resistance (5–7). As a result, research has broadened from traditional mechanisms like DNA damage responses to include metabolic adaptations that help tumors survive after irradiation (8). Among these changes, elevated lactate production caused by the Warburg effect has become a particularly notable feature (9, 10).

For a long time, lactate was regarded primarily as a metabolic waste product (11). This perspective has now been substantially revised. New evidence shows that lactate has multiple roles in tumor biology: it can act as a carbon source for oxidative metabolism, serve as a signaling metabolite that influences cellular behavior, and provide a biochemical context for post-translational regulation through lactylation (12). Notably, the discovery of histone lysine lactylation in 2019 introduced a new viewpoint on the connection between metabolism and epigenetic control (13). These developments support the view that lactate and lactylation may contribute to tumor radioresistance, but the strength of evidence differs substantially across metabolic, epigenetic, immune, and RNA-regulatory mechanisms.

**Figure 1:**
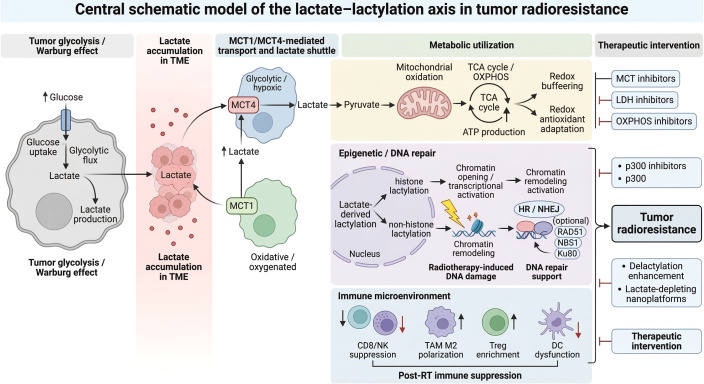
Central schematic model of the lactate–lactylation axis in tumor radioresistance. The image summarizes the proposed mechanisms through which lactate metabolism and lactylation may contribute to tumor radioresistance. Enhanced glycolytic flux promotes lactate accumulation in the tumor microenvironment, where lactate and protons are redistributed through context-dependent MCT1/MCT4-mediated transport and metabolic shuttling between distinct tumor cell populations. Lactate can then support mitochondrial oxidation, TCA cycle activity, OXPHOS, redox adaptation, and stress-response signaling, without implying that lactate-derived ATP is the sole driver of resistance. In addition, lactate-derived lactyl-CoA may provide a biochemical context for histone and non-histone lactylation, which has been implicated in chromatin remodeling, transcriptional activation of DDR-associated genes, and functional regulation of selected DNA repair proteins. Lactate-rich and acidic niches can also contribute to post-radiotherapy immune suppression by impairing CD8^+^ T-cell and NK-cell function, promoting TAM M2 polarization and Treg enrichment, and reducing DC activity. These metabolic, epigenetic, and immunological effects may collectively favor tumor persistence after irradiation. Potential therapeutic interventions include MCT, LDH, and OXPHOS inhibitors, indirect p300/CBP-related modulation, delactylation-related approaches, and lactate-depleting nanoplatforms.

Against this background, this review examines how lactate metabolism and lactylation may contribute to radioresistance in solid tumors. We first outline lactate production, bidirectional transport, clearance, and accumulation within the tumor microenvironment. We then discuss metabolic mechanisms, including oxidative substrate use, redox adaptation, and DNA damage repair, while avoiding an exclusively ATP-centered interpretation. Next, we review lactylation-mediated chromatin and non-histone protein regulation, followed by immune remodeling after radiotherapy. RNA-processing mechanisms are considered separately as emerging and incompletely causal links. Finally, we summarize therapeutic strategies and future research priorities, emphasizing the distinction between established, emerging, and hypothetical mechanisms (**Figure 1**).

### 1.1 Literature search strategy and evidence framing

This narrative review was prepared from literature searches of PubMed, Web of Science, Scopus, and Google Scholar using combinations of the terms lactate, lactylation, radiotherapy, radioresistance, DNA damage repair, tumor microenvironment, immune suppression, MCT, LDH, OXPHOS, m6A, and RNA processing. Priority was given to mechanistic studies in irradiated tumor models, followed by cancer models without irradiation, immune-cell studies, biochemical or structural studies, and clinically oriented reports. Throughout the review, we distinguish direct evidence from irradiated tumor systems from mechanisms inferred from related models and from hypothetical future directions. The search was last updated in June 2026.

## 2 Lactate metabolism: shaping the radioresistant phenotype

### 2.1 Classical concepts of lactate production and clearance

**Figure 2:**
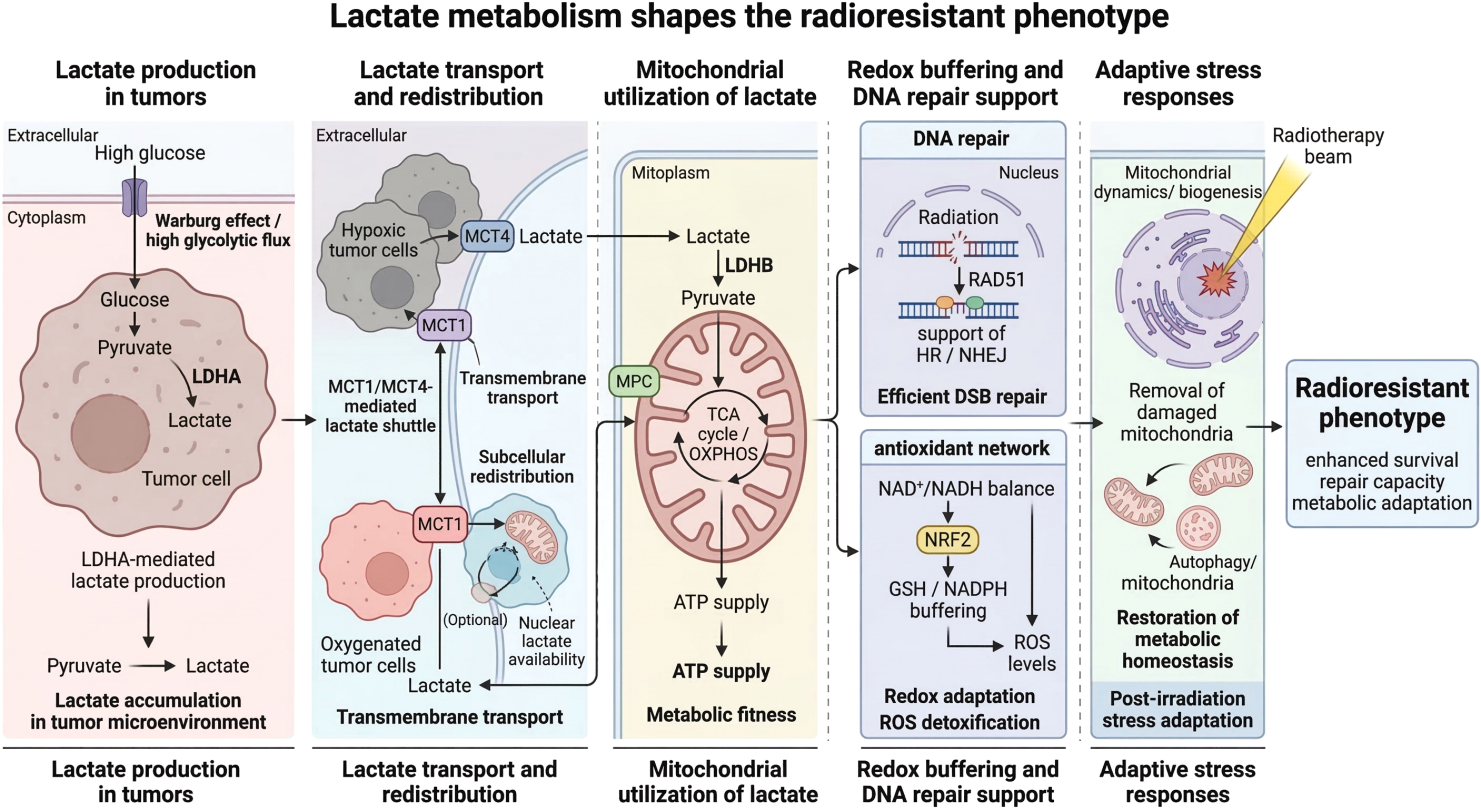
Schematic overview of how lactate metabolism may shape the radioresistant phenotype. The image summarizes the proposed mechanisms by which lactate metabolism contributes to tumor adaptation after irradiation. Increased glycolytic flux enhances LDHA-dependent lactate production and can promote accumulation of both lactate anions and protons in the tumor microenvironment. Lactate transport through MCT1/MCT4 is bidirectional and gradient-dependent, although MCT1 is often associated with uptake in oxidative cells and MCT4 with export in high-glycolytic cells because of their kinetic properties and cellular context. Lactate can be oxidized after uptake to support mitochondrial metabolism, TCA cycle activity, OXPHOS, and ATP production, but this operates alongside redox buffering through NAD^+^/NADH balance, NRF2-related antioxidant signaling, and GSH/NADPH-dependent detoxification pathways. These processes may help support post-irradiation DNA repair and reduce excessive oxidative damage. In addition, lactate may facilitate adaptive stress responses, including mitochondrial remodeling and autophagy/mitophagy, thereby contributing to metabolic recovery after radiotherapy. Together, these mechanisms may favor tumor persistence by enhancing survival, repair capacity, and metabolic flexibility.

Under physiological conditions, glucose is converted through glycolysis to pyruvate, which is primarily funneled into mitochondrial oxidation via the tricarboxylic acid (TCA) cycle in the presence of oxygen (**Figure 2**) (14). Under hypoxic conditions, however, pyruvate is preferentially reduced to lactate by lactate dehydrogenase A (LDHA), a process that regenerates NAD^+^ and sustains glycolytic flux (15). The resulting lactate anion and protons can be co-transported through monocarboxylate transporters (MCTs), enter the circulation, and are either recycled by the liver through gluconeogenesis in the Cori cycle or reutilized by highly oxidative tissues, particularly cardiac muscle and oxidative skeletal muscle fibers (16). In normal physiology, lactate concentrations in blood and interstitial fluids are typically maintained at approximately 1–3 mM (12, 17). This reflects a dynamic balance between lactate production, transport, and clearance, preventing its excessive accumulation under homeostatic conditions.

### 2.2 Lactate accumulation in the tumor microenvironment

A defining metabolic feature of many cancers is their preference for high glycolytic flux even in the presence of oxygen, a phenomenon known as aerobic glycolysis or the Warburg effect (18). One major consequence of this metabolic shift is the substantial accumulation of lactate within the tumor microenvironment (TME) (19). In many solid tumors, intratumoral lactate concentrations can reach levels far exceeding those observed in normal tissues, and elevated lactate has been associated with aggressive tumor behavior, metastatic potential, and adverse clinical outcomes (20). Biochemically, lactate exists predominantly as a conjugate-base anion at physiological pH; extracellular acidosis is driven by proton accumulation, although lactate and protons are frequently co-exported through MCT-dependent symport during high glycolytic flux (21). Therefore, low-pH effects and lactate-anion signaling should be considered related but mechanistically distinct. At the same time, lactate is not simply discarded: it can be taken up and oxidized by neighboring, better-oxygenated tumor cells, thereby establishing a form of metabolic symbiosis known as the lactate shuttle (22). Importantly, elevated lactate has also been associated with poor radiotherapeutic response in several tumor settings (23, 24). Tumors with lower lactate production tend to be more radiosensitive, whereas tumors characterized by marked lactate accumulation are often more refractory to irradiation. Thus, lactate-rich and proton-rich tumor niches are hallmarks of metabolic reprogramming that can influence tumor behavior and treatment response through partly separable mechanisms (25).

### 2.3 Transmembrane transport and subcellular redistribution of lactate

The transport of lactate across cellular and subcellular membranes is mediated primarily by members of the MCT family (26). MCT-mediated transport is bidirectional and depends on transmembrane lactate and proton gradients rather than on intrinsically one-way transporter function (27). The functional distinction between MCT isoforms mainly reflects kinetic properties and cellular context: MCT1 has relatively high affinity for monocarboxylates and can support lactate uptake in oxidative cells at lower extracellular lactate concentrations, whereas MCT4 has lower affinity but greater capacity and is well suited to high-glycolytic or hypoxic cells with elevated intracellular lactate production (28). In tumors, MCT1 and MCT4 are often co-expressed, enabling spatially organized lactate trafficking between metabolic niches. Lactate released from hypoxic tumor cells through MCT4-favored export can be taken up by better-oxygenated cells through MCT1-favored transport and oxidized as a fuel, thereby alleviating metabolic stress in oxygen-deprived regions while sustaining overall tumor growth (29, 30). Concurrent MCT-dependent proton co-transport contributes to extracellular acidification, which can suppress effector T-cell and NK-cell activity and promote macrophage polarization toward an immunosuppressive M2-like state. For these reasons, MCT1 and MCT4 are increasingly viewed as context-dependent mediators of metabolic symbiosis and immune escape, and their elevated expression is often associated with poor prognosis (31).

Beyond plasma membrane transport, lactate may also undergo subcellular redistribution (32). Evidence suggests that lactate-derived carbons can be transferred from the cytosol into mitochondrial metabolism, where lactate is converted to pyruvate and subsequently oxidized (33). In parallel, elevated intracellular lactate has been proposed to provide a permissive biochemical environment for nuclear lactylation, including histone lactylation under conditions such as hypoxia or enhanced glycolytic flux (34). Although the precise compartmental regulation of intracellular lactate handling remains incompletely defined, available evidence supports the view that MCT-mediated transport is a central mechanism governing lactate redistribution across cells and intracellular metabolic networks, thereby enabling flexible lactate utilization in tumors (35, 36).

### 2.4 Lactate as a carbon source for the TCA cycle and oxidative phosphorylation

Lactate has traditionally been viewed as a fallback metabolite generated when oxidative glucose metabolism is constrained. This concept has been substantially revised by isotope-tracing studies showing that lactate can function as a major oxidative carbon source and directly contribute to the TCA cycle and mitochondrial oxidative phosphorylation (OXPHOS) (37). In human lung cancer xenografts simultaneously infused with ^13^C-glucose and ^13^C-lactate, lactate-derived carbon contributed more prominently to TCA cycle intermediates than glucose-derived carbon, indicating that lactate can serve as an efficient substrate for oxidative metabolism in tumors (37). Similar observations have been reported in normal tissues as well as in genetically engineered models of lung and pancreatic cancer, supporting the broader relevance of lactate oxidation across biological contexts (38, 39).

Mechanistically, lactate may enter cells through MCT-facilitated transport, be converted to pyruvate by LDHB, and then be transported into mitochondria via the mitochondrial pyruvate carrier (MPC), where it enters the TCA cycle (40). More recent studies further suggest that lactate may influence mitochondrial function beyond its role as a conventional metabolic substrate. Under conditions of extracellular lactate accumulation, lactate has been reported to enhance electron transport chain activity and OXPHOS output, potentially acting as a signaling cue that promotes oxidative metabolic adaptation. Intriguingly, even D-lactate, an isomer not readily metabolized by LDH, has been reported to stimulate mitochondrial ATP production and suppress glycolytic activity in some experimental systems (41, 42). Although the mechanistic interpretation of these findings requires further clarification, they collectively support the notion that lactate is not a terminal waste product, but rather a context-dependent metabolic and signaling molecule that can contribute to tumor bioenergetics without being the sole energetic substrate available to cancer cells.

### 2.5 Lactate-supported OXPHOS: ATP supply, redox buffering, and metabolic flexibility

Maintenance of ATP availability is important for tumor cells to sustain post-irradiation repair processes and survival, but cancer cells can draw on multiple substrates, including glucose, glutamine, fatty acids, and lactate (43). By promoting oxidative metabolism in selected contexts, lactate may help irradiated tumor cells meet energetic demands, yet it should not be regarded as the universal or primary ATP source in all tumors. As discussed above, lactate can contribute to TCA cycle activity and mitochondrial oxidative flux. Even under oxygen-replete conditions, exogenous lactate has been shown to increase mitochondrial ATP synthesis while reducing reliance on glycolysis, thereby shifting the overall metabolic phenotype toward greater dependence on OXPHOS (44, 45). Cells with strong lactate-utilizing capacity may therefore maintain a more stable energy supply under radiation-induced metabolic stress, while other tumors may rely more heavily on alternative fuels (46).

Enhanced OXPHOS, however, is closely linked to changes in intracellular reactive oxygen species (ROS). Excessive ROS can amplify DNA damage and increase radiosensitivity, whereas moderate ROS may trigger adaptive antioxidant responses (47). In this context, lactate appears to act less as a simple ATP source than as part of a broader metabolic adaptation. Lactate exposure has been reported to induce a modest ROS burst and trigger adaptive antioxidant responses, including NRF2-associated programs, in some experimental systems (48, 49). This hormetic effect has been observed in neuroblastoma cells and experimental model organisms. In tumors, lactate-associated OXPHOS may therefore serve dual functions: supporting energetic homeostasis and promoting redox-adaptive defenses that reduce the accumulation of lethal ROS and DNA damage following irradiation (50). Taken together, lactate may increase tumor radioresistance by coordinating bioenergetic support, redox adaptation, and stress-response signaling.

### 2.6 Lactate-supported metabolic flexibility, redox adaptation, and DNA double-strand break repair

DNA double-strand breaks (DSBs) are the most lethal lesions induced by ionizing radiation, and enhanced DSB repair through homologous recombination (HR) or non-homologous end joining (NHEJ) is a well-recognized mechanism of tumor radioresistance (51). A lactate-rich metabolic environment appears to favor more efficient DSB repair, but the underlying explanation is likely multifactorial. Lactate-derived oxidative metabolism may support energy-intensive repair processes, including nucleotide synthesis, chromatin remodeling, and ligation reactions (23). At the same time, lactate-mediated redox buffering, histone lactylation, chromatin-based gene expression, HIF-1α-linked stress signaling, and immune modulation may provide equally important or more direct explanations for the association between lactate accumulation and radioresistance (46, 52).

Experimental evidence supports parts of this concept. In non-small cell lung cancer models, pharmacologic inhibition of LDHA reduced lactate production and significantly impaired post-irradiation DNA damage repair, leading to greater residual DNA breaks and diminished clonogenic survival (53). These effects were associated with insufficient ATP availability and secondary ROS accumulation, but they should not be interpreted as proving that lactate-derived ATP is the primary driver of radioresistance. Lactate treatment has also been reported to upregulate DNA repair-related genes, including RAD51, a central mediator of HR, suggesting that transcriptional and epigenetic adaptation may operate alongside metabolic support (54, 55). Conversely, suppression of lactate production or transport, for example through LDHA inhibition or MCT blockade, can reduce repair-associated gene expression and enhance radiosensitivity (27). Overall, lactate-supported radioresistance is better viewed as a layered model involving metabolic flexibility, redox control, chromatin regulation, and DDR signaling rather than as a single lactate-oxidation-ATP pathway.

### 2.7 Lactate and antioxidant networks: NAD^+^/NADH, glutathione, and NRF2

The cytotoxicity of radiotherapy depends largely on ionizing radiation-induced ROS, which cause irreversible damage to DNA, proteins, and membrane lipids (56). Accordingly, the strengthening of antioxidant defenses is a key route through which tumor cells acquire radioresistance. High-lactate conditions may reinforce these defenses through multiple layers of redox regulation (57).

First, lactate metabolism is closely linked to the cellular NAD^+^/NADH balance (58). The LDHA-catalyzed conversion of pyruvate to lactate consumes NADH and regenerates NAD^+^, whereas the reverse LDHB-catalyzed reaction generates NADH from lactate oxidation (59). This reversibility positions lactate metabolism as an important buffer for intracellular redox balance. Adequate NAD^+^ availability is essential for PARP-mediated repair of DNA single-strand breaks and for the activity of sirtuin deacetylases involved in chromatin stability and stress responses (60, 61). By supporting LDH-dependent redox cycling, lactate may help preserve metabolic homeostasis and repair capacity under radiation-induced oxidative stress (40, 53). Conversely, disruption of lactate production may perturb the NAD^+^/NADH ratio and impair downstream antioxidant and repair programs.

Second, lactate may activate the NRF2 antioxidant pathway. As noted above, modest ROS induction by lactate can trigger NRF2 activation, leading to transcriptional upregulation of antioxidant and detoxification genes, including those involved in glutathione synthesis and peroxide clearance (33). In neuroblastoma cells, lactate exposure has been shown to induce low-level ROS signaling, promote NRF2 nuclear translocation, and enhance expression of multiple stress-response genes (48, 49). Under radiotherapy, where ROS production can overwhelm cellular defenses, tumor cells preconditioned by high lactate may therefore be better equipped to detoxify oxidative stress and survive irradiation.

Third, lactate may influence glutathione (GSH) metabolism and related reductive pathways (62). GSH is a major intracellular antioxidant, and the balance between reduced glutathione (GSH) and oxidized glutathione (GSSG) is sustained by reducing equivalents such as NADPH. Several metabolic pathways linked to lactate utilization, including the pentose phosphate pathway (PPP) and lactate-pyruvate cycling, are closely connected to NADPH homeostasis (63). Under glucose-limited conditions, tumor cells can use lactate together with glutamine to sustain NADPH production and maintain reductive capacity (64). In particular, lactate has been reported to promote PPP activity by enhancing the function of glucose-6-phosphate dehydrogenase (G6PD), the rate-limiting enzyme of this pathway (65). One proposed mechanism is that lactate binds glutathione S-transferase P1 (GSTP1), thereby preventing inhibitory phosphorylation of G6PD and increasing PPP flux, NADPH generation, and antioxidant resistance (43). Interruption of this lactate–GSTP1–G6PD signaling axis reduces antioxidant capacity and restrains tumor growth in experimental models (64). Collectively, these findings suggest the possibility that elevated lactate supports radioresistance by strengthening antioxidant networks through coordinated effects on NAD^+^/NADH balance, NRF2 activation, and GSH/NADPH^-^dependent redox buffering.

### 2.8 Lactate, mitochondrial dynamics, and autophagy in post-irradiation survival

Mitochondria play a dual role in the cellular response to radiation: they generate ATP needed for survival and repair, but they are also a major source of ROS that can amplify damage (66). To maintain mitochondrial function and prevent excessive oxidative stress, cells rely on mitochondrial dynamics, including fission and fusion, as well as selective autophagic removal of damaged mitochondria (67). Emerging evidence suggests that lactate accumulation may influence mitochondrial remodeling and autophagic responses, which could in turn affect tumor cell adaptation after irradiation (68).

In mitochondrial dynamics, high lactate has been associated with increased mitochondrial fission in some pathological contexts. For example, lactate has been shown to activate ERK/DRP1 signaling and promote excessive mitochondrial fragmentation in fibrotic models (69). Although these findings derive from non-tumor systems, they raise the possibility that lactate may also modulate mitochondrial remodeling in cancer cells. Controlled mitochondrial fission may facilitate the segregation and removal of damaged mitochondria after irradiation, thereby limiting ROS overload and preserving mitochondrial quality (70). At the same time, lactate has been linked to activation of mitochondrial biogenesis-related programs, including transcriptional regulators such as PGC-1α, suggesting a potential role in restoring oxidative capacity after stress (71). Such effects could enable tumor cells to re-establish energetic homeostasis after radiotherapy-induced mitochondrial injury.

Lactate has also been implicated in the regulation of autophagy, a process that is frequently activated under hypoxia and metabolic stress to remove damaged organelles and recycle intracellular substrates (72). Elevated lactate has been reported to stimulate autophagic activity, potentially through signaling pathways involving PI3K-C3/Vps34 and related autophagosome-forming machinery (73). More recently, it has been proposed that lactylation may directly connect glycolytic activity to autophagy by modifying proteins involved in autophagic regulation. Although the relevant substrates and mechanisms remain incompletely defined, this concept is particularly relevant to radioresistance (74). Autophagy can facilitate the clearance of radiation-damaged mitochondria and other injured cellular components, thereby reducing cytotoxic stress and supplying recyclable metabolites that support survival (75). Indeed, inhibition of autophagy has been shown to enhance radiation-induced cell death in multiple settings. Thus, lactate-mediated reinforcement of mitochondrial adaptation and autophagic activity may represent an additional metabolic layer that enables tumor cells to endure irradiation.

## 3 Lactylation: an emerging epigenetic bridge in the DNA damage response

### 3.1 Discovery and conceptual definition of lactylation

**Figure 3:**
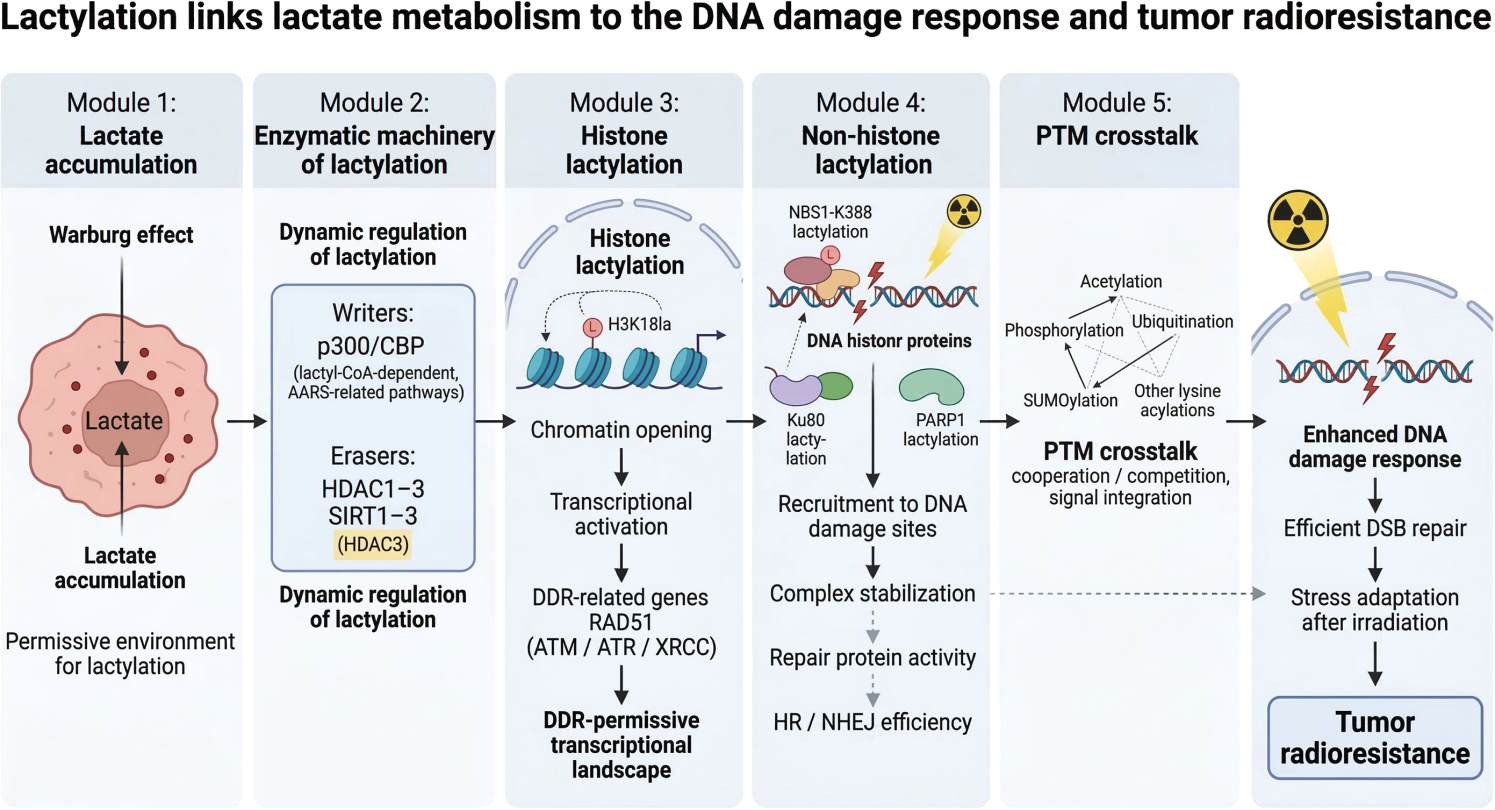
Schematic overview of how lactylation may connect lactate metabolism to DNA damage response regulation and tumor radioresistance. Enhanced glycolysis and lactate accumulation may create a permissive biochemical context for protein lactylation through lactyl-CoA or related activated acyl intermediates. The current enzymatic framework of lactylation includes p300/CBP-associated promiscuous acyltransferase activity and delactylase activity attributed to class I HDACs and selected sirtuins, although this machinery remains incompletely defined and no dedicated lactyltransferase has been definitively established. Histone lactylation may support a transcriptionally permissive chromatin state and promote expression of DDR-related genes, whereas non-histone lactylation has been implicated in the regulation of selected repair proteins, including NBS1 and other candidate DDR factors. In parallel, lactylation may function within a broader PTM network involving acetylation, phosphorylation, ubiquitination, SUMOylation, and other lysine acylations. Through these chromatin-based, protein-level, and PTM-crosstalk mechanisms, lactylation may contribute to enhanced DNA damage responses, more efficient double-strand break repair, post-irradiation adaptation, and tumor radioresistance. Dashed arrows denote hypothetical, context-dependent, or incompletely validated mechanisms.

In 2019, Zhang and colleagues reported lysine lactylation (Kla) as a previously unrecognized post-translational modification, thereby expanding the landscape of metabolite-linked protein regulation (**Figure 3**) (13). In inflammatory macrophages, they identified lactylated lysine residues on histone H3, including H3K18la, and demonstrated that this modification is derived from lactate and can be catalyzed by the histone acetyltransferase p300 through covalent transfer of a lactyl group to the epsilon-amino group of lysine residues (11, 76, 77). Structurally, lactylation belongs to the broader family of short-chain acyl modifications and introduces a distinct chemical moiety capable of neutralizing lysine charge, altering local hydrophobicity, changing protein conformation, and reshaping molecular interactions (78). As a result, lactylation has the potential to regulate cellular function at multiple levels.

At the chromatin level, histone lactylation is generally thought to neutralize the positive charge of lysine residues, thereby loosening local chromatin structure and facilitating transcriptional activation (79). In the original study, increased H3K18la promoted the expression of genes associated with macrophage M2 polarization, such as Arg1, supporting the concept that lactylation can function as a molecular interface between metabolic state and transcriptional output (13). Since then, lactylation has been detected not only on histones but also on a growing number of non-histone proteins, suggesting that its biological influence extends beyond chromatin regulation (80). Collectively, the discovery of lactylation has broadened the conceptual framework of epigenetic and post-translational regulation by showing that a metabolic product long regarded as biologically secondary can directly participate in shaping cellular phenotypes under pathological conditions, including cancer.

### 3.2 Enzymatic regulation of lactylation: writers and erasers

Like other reversible post-translational modifications, lactylation appears to be controlled by a dynamic balance between activated acyl-donor availability, enzymes that install the modification, and enzymes that remove it. Free lactate cannot be directly conjugated to proteins; it must first be converted into an activated lactyl-CoA or lactate-derived acyl intermediate through metabolic pathways that may involve acyl-CoA synthetases or related enzymes (77). Current evidence suggests that p300/CBP, best known as histone acetyltransferases, can catalyze lactylation under conditions in which lactyl-CoA is available, but this activity is best interpreted as promiscuous acyltransferase activity rather than proof of a dedicated lactyltransferase (76). The balance between acetyl-CoA and lactyl-CoA pools may therefore shape whether lysine acetylation or lactylation predominates at shared or nearby sites.

The identity of lactylation “erasers” has also begun to emerge. Several class I histone deacetylases, particularly HDAC1–3, as well as class III NAD^+^-dependent deacetylases such as SIRT1–3, have been reported to possess delactylase activity (81). Among these, HDAC3 has been highlighted as an efficient regulator of both histone and non-histone lactylation (82). Pharmacologic inhibition of HDAC activity can therefore lead to accumulation of lactylation marks, consistent with the idea that at least part of the deacetylase machinery also functions in lactylation turnover. In the context of DNA damage signaling, HDAC3 has been implicated as a delactylase for NBS1, and its loss results in increased NBS1 lactylation (23). Taken together, currently available data indicate that lactylation is regulated by a partially overlapping but not identical enzymatic network relative to acetylation. However, the lactylation machinery remains incompletely defined. Whether there are more substrate-selective lactyltransferases or dedicated delactylases, and how these enzymes operate across different cellular contexts, remain important unresolved questions.

### 3.3 Histone lactylation and transcriptional activation of DNA damage response genes

Histone lactylation may contribute to radioresistance by reshaping chromatin accessibility and transcriptional programs associated with DNA damage response (DDR), survival, and stress adaptation (83). A growing body of evidence suggests that genes involved in DNA repair and cell survival can be transcriptionally upregulated in lactate-rich environments, raising the possibility that histone lactylation contributes to this process (84). In tumor cells, as in macrophages, lactate-driven chromatin remodeling appears capable of inducing cell-state-specific gene expression programs. For example, lactate exposure has been reported to increase the expression of multiple DNA repair-associated genes, and elevated lactylation has been linked to increased RAD51 expression, a central mediator of homologous recombination (55). Because RAD51 overexpression is frequently associated with efficient repair of radiation-induced DNA double-strand breaks and reduced radiosensitivity, this observation is mechanistically relevant (84, 85).

One plausible model is that lactate accumulation promotes histone lactylation at promoters or enhancers of DNA repair-related genes, thereby creating a more transcriptionally permissive chromatin environment (13). Supporting this concept, glioma stem-like cells with high lactate output have been reported to display elevated histone lactylation at loci associated with DNA repair and cell-cycle regulation, accompanied by increased expression of these genes and enhanced tolerance to genotoxic stress (86). Conversely, suppression of global lactylation has been associated with downregulation of repair-related transcripts and greater sensitivity to irradiation. Beyond RAD51, other DDR regulators, such as ATM, ATR, and members of the XRCC family, may also be influenced by histone lactylation, although the evidence is less direct and remains incomplete (87). It is also important to note that lactylation likely does not operate in isolation: histone lactylation may cooperate or compete with other chromatin marks, including acetylation, in fine-tuning transcriptional outputs. Thus, while available findings support the idea that histone lactylation can promote a DDR-permissive transcriptional landscape, the extent to which this mechanism is causal, tumor-type-specific, or broadly generalizable remains unclear.

### 3.4 Emerging evidence that non-histone lactylation modulates DNA repair function

In addition to histones, lactylation has been identified on numerous non-histone proteins, including metabolic enzymes, transcription factors, and DNA repair factors themselves. These findings are particularly important because they raise the possibility that lactylation can regulate DDR not only indirectly through transcription, but also directly at the level of repair protein function. One of the most compelling examples is NBS1, a component of the MRE11-RAD50-NBS1 (MRN) complex that is essential for the early sensing and processing of DNA double-strand breaks (23). A recent study showed that lactate accumulation promotes lactylation of NBS1 at lysine 388, a modification installed by Tip60 and removed by HDAC3. Because lysine residues often contribute to electrostatic contacts, charge neutralization at specific sites may alter the recruitment, stability, or DNA-damage-site retention of repair complexes. Lactylated NBS1 displayed enhanced recruitment to sites of DNA damage and improved stabilization of the MRN complex, thereby facilitating DNA damage recognition and downstream signaling (23). Disruption of lactate production or mutational abrogation of the lactylation site impaired homologous recombination efficiency and reduced resistance to genotoxic stress, although the extent to which this mechanism generalizes across radiotherapy models requires further validation.

Additional evidence suggests that non-histone lactylation may also influence non-homologous end joining (NHEJ). In radioresistant lung cancer cells, multiple lactylation sites have been identified on Ku80 (XRCC5), and lactylation inhibition has been associated with reduced Ku70/80-dependent end-joining efficiency and increased radiosensitivity (88). A plausible biophysical mechanism is that lactylation adds a bulky acyl group and neutralizes positively charged lysines that normally help repair proteins engage the negatively charged DNA phosphate backbone. For Ku70/80 and PARP1, whose recruitment to damaged DNA depends heavily on electrostatic contacts and domain-level DNA affinity, site-specific lactylation could alter DNA-end binding, complex assembly, or recruitment of downstream effectors such as DNA-PK. PARP1 has also been reported to undergo lactylation in non-small cell lung cancer cells, where this modification appears to alter its interaction with PKM2 and support tumor cell survival, although this effect may be functionally separable from canonical PARP1-mediated DNA repair (74, 86). Taken together, these studies begin to outline a functional blueprint in which lactylation can directly optimize the activity, assembly, or localization of selected repair proteins. However, direct structural evidence remains sparse, and broader conclusions about non-histone lactylation in radioresistance should therefore be made cautiously.

### 3.5 Crosstalk between lactylation and canonical post-translational modifications

As an emerging post-translational modification, lactylation is unlikely to act independently of the broader PTM landscape. Instead, it appears to participate in a complex network of cooperation and competition with established modifications such as acetylation, phosphorylation, ubiquitination, SUMOylation, and other lysine acylations (13). The most immediate relationship is with acetylation, because both are short-chain acyl modifications that frequently target the same lysine residues. As such, lactylation and acetylation can be mutually exclusive at specific sites. Histone H3K18, for example, may undergo either acetylation or lactylation depending on metabolic context, and shifts in intracellular lactate availability may alter the balance between these marks, thereby changing transcriptional outcomes (86). Conversely, manipulation of acetyltransferases or deacetylases can secondarily influence lactylation, consistent with the partial overlap between the enzymatic machinery governing these modifications.

Lactylation may also intersect with other lysine-centered modifications that regulate protein stability, localization, or signaling. Occupation of a lysine residue by a lactyl group could, in principle, prevent ubiquitination or SUMOylation at the same site, thereby altering turnover or subcellular trafficking of the modified protein (89). This possibility is particularly relevant for DDR proteins, many of which rely on dynamically coordinated PTM switches to orchestrate damage recognition and the choice of repair pathways (90, 91). In addition, lactylation may influence phosphorylation-dependent signaling indirectly by altering local protein conformation or protein-protein interactions (92). More broadly, because lactylation arises from metabolic state, it may serve as a biochemical layer through which redox status, carbon flux, and signaling pathways converge. These crosstalk relationships likely contribute to the pleiotropic effects of lactylation on stress adaptation and therapeutic resistance. However, systematic dissection of these PTM interactions remains in its early stages, and many of the relevant mechanisms in irradiated tumors have yet to be fully resolved (91).

### 3.6 Emerging, causality-limited links between lactate-lactylation signaling and post-transcriptional gene regulation

Although lactylation has been most commonly discussed in relation to chromatin remodeling and protein function, its potential connection with post-transcriptional gene regulation is increasingly relevant in cancer biology. Gene expression programs that support radioresistance are not determined solely by transcriptional activation; they are also shaped by RNA processing steps, including RNA modification, alternative splicing, mRNA stability, translational control, and non-coding RNA-mediated regulation (93). These processes can rapidly remodel transcripts involved in DNA damage repair, redox adaptation, immune evasion, and cell survival after irradiation. However, most links between lactate-lactylation signaling and RNA processing remain indirect in radiotherapy settings.

Among these mechanisms, m6A RNA modification provides one of the most plausible but still incompletely causal links between lactate-associated signaling and RNA-level gene regulation. Recent evidence suggests that lactylation-dependent regulation of METTL3 in myeloid suppressive populations may enhance m6A-dependent stabilization of transcripts associated with immunosuppressive programs (94). At present, it remains unresolved whether METTL3 lactylation directly changes METTL3 conformation, RNA-binding affinity, substrate selection, or interaction with m6A writer-complex partners, or whether it reflects a broader metabolic-stress state that co-occurs with immunosuppression (95). In the context of radiotherapy, such regulation could affect the persistence of transcripts encoding immune-suppressive mediators, stress-response proteins, or DNA repair factors, but direct causal evidence in irradiated tumor models remains limited.

Non-coding RNAs may provide another interface between lactate metabolism and gene expression regulation. Multiple miRNAs, lncRNAs, and circRNAs have been implicated in the regulation of glycolysis, LDHA expression, MCT-mediated lactate transport, DNA repair capacity, and radiosensitivity (96). Conversely, lactate-driven transcriptional and epigenetic remodeling may alter the production or function of non-coding RNAs that participate in therapy resistance (97). Nevertheless, direct evidence connecting lactylation to specific RNA processing events in irradiated tumors remains limited. Therefore, RNA processing should currently be positioned as a future research direction requiring lactyl-proteomics, RNA-seq, m6A-seq, splicing analysis, structural assessment of RNA-binding proteins, and functional rescue experiments to distinguish correlation from causality.

## 4 The lactate–lactylation axis in the post-radiotherapy immune microenvironment

### 4.1 Lactate-anion signaling and proton-driven acidosis suppress post-irradiation effector T-cell and NK-cell function

**Figure 4:**
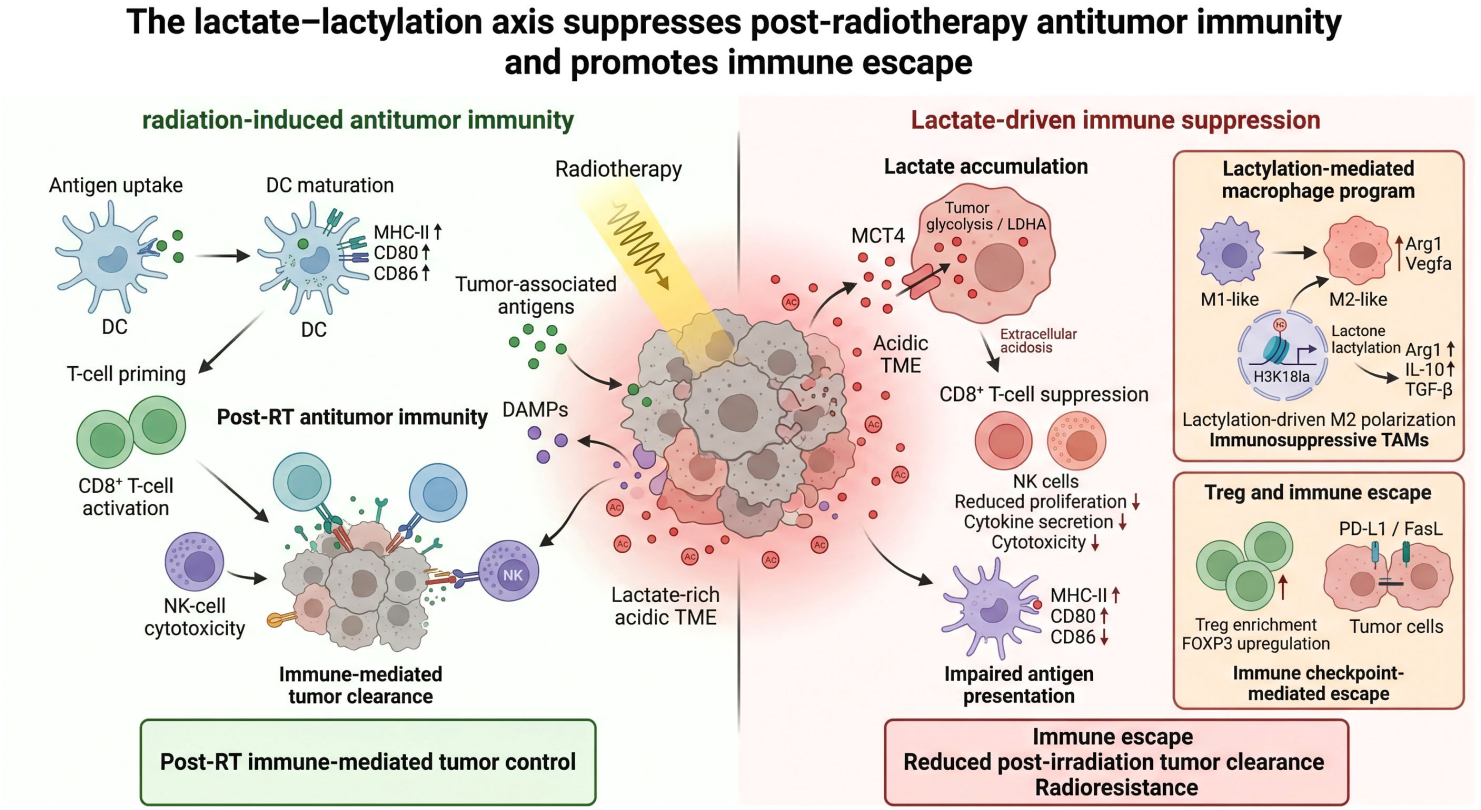
Schematic overview of how the lactate–lactylation axis may counteract radiotherapy-induced antitumor immunity. The figure illustrates the balance between radiation-induced immune activation and lactate-rich or acidic immune suppression in the post-radiotherapy tumor microenvironment. On the left, radiotherapy promotes tumor antigen and DAMP release, supporting DC maturation, T-cell priming, NK-cell activation, and immune-mediated tumor clearance. On the right, tumor glycolysis and LDHA activity increase lactate generation and MCT-dependent lactate/proton co-export, creating related but mechanistically distinct lactate-anion signaling and proton-driven acidosis. These conditions suppress effector lymphocyte function and impair antigen presentation. Lactate and lactylation may further promote macrophage polarization toward immunosuppressive M2-like states, favor Treg enrichment, and support tumor-cell immune escape programs. Through these mechanisms, the lactate-rich tumor microenvironment may attenuate the immunogenic effects of radiotherapy and reduce post-irradiation tumor clearance, thereby contributing to radioresistance.

Anti-tumor immunity is an important contributor to the therapeutic efficacy of radiotherapy, particularly through radiation-induced immune activation and the subsequent elimination of residual tumor cells (**Figure 4**). However, lactate-rich and acidic tumor niches can substantially weaken this component of the radiotherapeutic response (98). Mechanistically, these effects should be bifurcated: proton-driven extracellular acidosis can impair immune-cell function through low-pH biophysics, whereas the lactate anion can influence metabolism and signaling through transporters and receptors (99). Under acidic conditions, cytotoxic T cells exhibit reduced proliferation, cytokine production, and tumor-killing activity, while NK-cell cytotoxicity is likewise diminished. Elevated glycolytic flux and LDHA activity can increase intracellular lactate generation and MCT-dependent lactate/proton co-export, thereby facilitating tumor progression by weakening immune surveillance (100).

In addition to proton-driven extracellular acidosis, lactate anions may directly interfere with immune-cell metabolism and signaling. T cells express lactate-sensitive transport and signaling systems, including monocarboxylate transporters and G protein-coupled receptors such as GPR81 (101). Excess lactate uptake can disrupt intracellular pH and metabolic homeostasis, impairing effector function and reducing cytokine secretion. This is particularly relevant following radiotherapy, when irradiated tumor cells release tumor-associated antigens that would otherwise be expected to stimulate anti-tumor immune responses (98). In a lactate-rich and low-pH environment, however, dendritic cell antigen-presenting capacity is diminished, co-stimulatory signaling is weakened, and efficient T-cell priming becomes more difficult to achieve. Lactate may also blunt innate immune cooperation by limiting monocyte recruitment and suppressing the secretion of pro-inflammatory cytokines such as TNF-α and IL-6 (102). Taken together, these effects suggest that lactate accumulation and acidosis can reduce post-irradiation immune-mediated tumor clearance through overlapping but distinct mechanisms.

### 4.2 M2 macrophage polarization and lactylation-driven transcriptional programs

Tumor-associated macrophages (TAMs) are major determinants of whether the tumor immune microenvironment adopts an anti-tumor or pro-tumor phenotype (103). Broadly speaking, M1-like macrophages support antigen presentation and cytotoxic immune activation, whereas M2-like macrophages are associated with tissue repair, immune suppression, and tumor progression (104, 105). Elevated lactate concentrations in tumors have been shown to promote polarization of TAMs toward an M2-like state (104, 106). Mechanistically, lactate-driven histone lactylation appears to play a key role in this process. In the landmark study describing histone lactylation, high-lactate conditions induced H3 lactylation, including H3K18la, in macrophages and promoted the transcription of M2-associated genes such as Arg1 and Vegfa (13). This finding provided direct evidence that metabolic state can influence immune-cell fate through an epigenetic mechanism.

In the setting of radiotherapy, M2-polarized TAMs may undermine treatment efficacy in several ways. These cells secrete immunosuppressive mediators such as IL-10 and TGF-β, which dampen immune responses against radiation-exposed tumor cells (104, 107). They can also suppress T-cell proliferation by depleting L-arginine via ARG1 expression. Clinically, high-lactate tumors have been associated with increased infiltration of CD163^+^ M2-like macrophages, and such macrophage-rich immunosuppressive niches are generally linked to poorer therapeutic responses (108). More recent studies further suggest that lactylation may extend beyond histones to regulate broader immunosuppressive programs. For example, lactylation-dependent regulation of METTL3 in myeloid suppressor populations has been proposed to enhance m6A-dependent stabilization of transcripts associated with immune suppression, providing a possible bridge between lactate-driven metabolic remodeling, RNA processing, and post-radiotherapy immune escape (95). Because this connection has not yet been rigorously established as a direct causal driver in irradiated tumor models, it should be interpreted as an emerging mechanism requiring further validation.

### 4.3 Radiation-induced antigen release versus lactate-driven immune evasion

Beyond its direct cytotoxic effects, radiotherapy can enhance tumor immunogenicity by promoting the release of tumor-associated antigens and danger-associated molecular patterns (DAMPs), thereby facilitating dendritic cell recruitment, antigen uptake, and T-cell priming (109). This process is often invoked to explain the immunostimulatory component of radiotherapy, including so-called *in situ* vaccination effects (4). However, high lactate levels may substantially attenuate this benefit and allow tumors to evade immune elimination even after irradiation.

One important mechanism is the suppression of dendritic cell maturation and function. In lactate-rich and acidic environments, dendritic cells display impaired differentiation, reduced antigen presentation, and lower expression of MHC class II molecules and co-stimulatory factors such as CD80 and CD86 (109). Lactate-anion signaling through receptors such as GPR81, together with metabolic constraints and proton-driven low-pH stress, can shift dendritic cells away from immune activation and toward more tolerogenic or pro-angiogenic states, including increased production of factors such as VEGF at the expense of cytokines that support Th1-polarized immunity (101). As a result, radiotherapy-induced antigen release may fail to translate into effective adaptive immune activation (109).

Lactate and acidosis also act selectively across lymphocyte subsets. Whereas effector T cells and NK cells are functionally inhibited under high-lactate or low-pH conditions, regulatory T cells (Tregs) appear comparatively more tolerant of acidic and lactate-rich environments and may even expand under such conditions (110). Hypoxia, lactate accumulation, and extracellular acidosis have been linked to increased expression of lineage-defining factors such as FOXP3, thereby supporting Treg persistence and suppressive activity (111). Treg enrichment can further restrain the activity of effector T cells and other immune populations, reinforcing a locally immunosuppressive niche (112). In addition, acidic and lactate-rich conditions may induce adaptive changes in tumor cells, including increased expression of immune checkpoint-related molecules such as PD-L1 and, in selected contexts, apoptosis-related immune-evasion pathways (113). Overall, these findings support the view that lactate-rich and proton-rich niches can counterbalance the immunogenic effects of radiotherapy by imposing metabolically and biophysically driven programs of immune evasion (114). This has important translational implications, particularly in the era of combined radiotherapy and immunotherapy, where overcoming lactate-associated immune suppression may be essential for maximizing therapeutic benefit.

## 5 Therapeutic targeting: from radiosensitization to translational barriers

### 5.1 Targeting lactate transport across membranes: MCT inhibitors

Inhibition of transmembrane lactate transport represents one of the most direct strategies to disrupt tumor lactate metabolism and enhance radiosensitivity (**Table 1**). By blocking lactate export and uptake, monocarboxylate transporter (MCT) inhibitors interfere with metabolic symbiosis within tumors and may simultaneously alter the biochemical and immune properties of the tumor microenvironment (29). Among these agents, the selective MCT1 inhibitor AZD3965 is the most clinically advanced representative (115). By inhibiting MCT1-mediated lactate transport, AZD3965 impairs both lactate influx and efflux in susceptible tumor cells, thereby disrupting lactate shuttling and constraining metabolic flexibility (116).

**Table 1:** Lactate and lactylation targeting strategies for tumor radiosensitization.

Strategy/representative inhibitor	Specific metabolic target	Clinical trial phase/status	Radiosensitizing rationale	Primary compensation mechanism/barrier
MCT inhibition: AZD3965; dual MCT concepts	MCT1-dependent lactate/H^+^ symport; context-dependent lactate import/export shaped by MCT4.	AZD3965: Phase I in advanced solid tumors/lymphoma; RT combinations remain preclinical.	Disrupts lactate shuttling, increases metabolic stress, and may reduce lactate-rich or low-pH immune suppression.	MCT4 upregulation and alternative lactate handling; response likely depends on low MCT4 expression.
LDH inhibition: oxamate; NHI-1/2; GSK2837808A; galloflavin	LDHA/LDHB-mediated lactate-pyruvate cycling and NAD^+^/NADH balance.	Mainly preclinical for radiosensitization; no mature RT clinical phase for listed LDH inhibitors.	Reduces lactate production or reutilization, perturbs redox buffering, and increases radiation-induced oxidative damage.	Isoform redundancy, LDHB/LDHA compensation, systemic metabolic toxicity, and alternative fuel use.
OXPHOS inhibition: phenformin; IACS-010759; metformin-related concepts	Mitochondrial complex I, oxidative ATP production, and redox adaptation.	IACS-010759: Phase I in AML/advanced solid tumors with toxicity limitations; RT combinations mainly preclinical.	Limits mitochondrial metabolic resilience after irradiation and may improve RT and immunotherapy responses.	Lactic acidosis, narrow therapeutic window, normal-tissue respiration dependence, and metabolic plasticity.
Indirect lactylation modulation: A-485; C646; delactylase-related concepts	p300/CBP promiscuous acyltransferase activity and lactyl-CoA/acetyl-CoA-dependent acyl pools.	Mechanistic/preclinical; no dedicated lactyltransferase-targeted clinical phase.	May dampen acylation-dependent DDR or stress-adaptive transcriptional programs.	Poor p300/CBP specificity; incomplete writer/eraser definition and substrate selectivity.
Lactate-depleting nanoplatforms: LOX nanoreactors; inhibitor-loaded particles	Local lactate consumption or tumor-activated metabolic disruption.	Proof-of-concept preclinical; no mature clinical RT phase.	Locally reduces lactate-rich immune suppression and may synergize with RT or anti-PD-1 therapy.	Manufacturing, biodistribution, reproducibility, and regulatory burden.
Rational combinations: RT + anti-PD-1; RT + PARPi; metabolic inhibitor combinations	Immune suppression, repair vulnerability, and metabolic compensation pathways.	Emerging translational/preclinical; clinical sequencing for lactate-axis RT combinations unresolved.	Combines metabolic, immune, and DDR vulnerabilities to widen therapeutic opportunities.	Timing, patient selection, immune-cell toxicity, and resistance adaptation.

Early-phase clinical evaluation has shown that AZD3965 is tolerable in patients with advanced solid tumors and lymphomas, with reversible electroretinographic changes and mild metabolic disturbances among the main reported toxicities (30). In preclinical tumor models, AZD3965 treatment increases intracellular lactate accumulation, perturbs metabolic homeostasis, and suppresses tumor growth (117). When combined with radiotherapy, MCT1 inhibition has demonstrated enhanced tumor control and prolonged survival in animal models (118). Several mechanisms may underlie this radiosensitizing effect. Blocking MCT1 disrupts lactate utilization by oxidative tumor cells, thereby weakening metabolic cooperation between hypoxic and oxygenated tumor regions (115). It may also promote intracellular acidification, impair glycolytic compensation, and increase oxidative stress, all of which can sensitize tumor cells to irradiation.

However, the therapeutic impact of MCT1 inhibition is highly context dependent. A major limitation is compensatory MCT4 expression, which can preserve lactate export and thereby reduce susceptibility to MCT1-targeted therapy (29). For this reason, MCT4 expression has emerged as a potential biomarker for patient selection: tumors with low MCT4 expression are generally considered more likely to respond to AZD3965, whereas MCT4-high tumors may require dual targeting strategies or alternative metabolic interventions (28). At present, no selective MCT4 inhibitor has entered routine clinical development, although non-selective MCT inhibitors and compounds reported to interfere with MCT4 function have shown preliminary activity in preclinical settings (119). Overall, MCT inhibition remains one of the most conceptually attractive lactate-targeting approaches for radiosensitization because it simultaneously affects tumor metabolism, acidosis, and immune suppression. Its ultimate clinical utility, however, will likely depend on biomarker-guided selection and rational strategies to overcome transporter redundancy.

### 5.2 Targeting lactate production and utilization: LDH inhibitors

Lactate dehydrogenase (LDH) sits at a central node of lactate metabolism and is therefore an appealing target for radiosensitization (120). Inhibition of LDH not only reduces lactate production or reutilization but can also disrupt NAD^+^/NADH cycling, alter glycolytic flux, impair ATP homeostasis, and increase oxidative stress (121). Through these combined effects, LDH inhibitors may weaken multiple metabolic adaptations that support tumor survival following irradiation.

Several LDHA-targeting agents, including oxamate, NHI-1/2 derivatives, and GSK2837808A, have shown radiosensitizing effects in preclinical models. *In vitro*, oxamate reduces ATP availability, increases ROS accumulation, and enhances radiation-induced DNA damage and apoptosis. In xenograft models, the combination of oxamate and radiotherapy has shown greater tumor growth inhibition than radiotherapy alone (122). More selective LDHA inhibitors, such as NHI compounds, have also demonstrated activity in glioblastoma models, where they appear to promote differentiation and apoptosis of tumor stem-like cells while enhancing sensitivity to radiotherapy and chemotherapy (123). GSK2837808A has additionally been linked to modulation of the tumor immune microenvironment; in pancreatic cancer-related models, LDH inhibition has been reported to reduce myeloid suppressive activity and improve T-cell-mediated anti-tumor responses in the setting of radiotherapy (124).

LDHB inhibition offers a related but mechanistically distinct approach by limiting lactate oxidation to pyruvate and thereby impairing lactate reutilization (125). Compounds such as galloflavin have shown potential in preclinical studies, but the field still lacks highly selective LDHB inhibitors with convincing translational trajectories. Broad-spectrum LDH inhibitors, including AT-101, have also been explored, although their pleiotropic effects complicate mechanistic interpretation (126). A major translational concern across this class is systemic toxicity. Because LDH is essential to normal metabolic physiology, global inhibition may increase the risk of lactate accumulation, redox imbalance, or broader metabolic adverse effects (127). Thus, despite encouraging preclinical radiosensitization data, the therapeutic window of LDH inhibition remains a major issue. Future progress may depend on tumor-selective delivery, better isoform targeting, and rational combination strategies, including pairing LDH inhibition with DNA repair-targeted therapies to exploit metabolically induced repair vulnerabilities.

### 5.3 Targeting mitochondrial oxidative phosphorylation: metabolic inhibitors in combination with radiotherapy

Given that enhanced oxidative phosphorylation (OXPHOS) can contribute to lactate-supported radioresistance, targeting mitochondrial respiration represents another attractive radiosensitization strategy. By limiting ATP production and altering redox homeostasis, OXPHOS inhibitors can reduce the metabolic resilience of tumor cells and potentially intensify the consequences of radiation-induced damage (50). Representative agents include mitochondrial complex I inhibitors such as phenformin and IACS-010759 (128).

Phenformin, a more potent analog of metformin, inhibits electron transport through mitochondrial complex I and thereby suppresses ATP generation (39). In preclinical studies, phenformin has shown the capacity to increase metabolic stress in tumors and, in some models, to enhance radiosensitivity, particularly in hypoxic or OXPHOS-dependent settings. IACS-010759, developed as a potent complex I inhibitor, has demonstrated anti-tumor activity across multiple models and has attracted particular interest in tumors with strong mitochondrial dependence (129). More recent work suggests that radiotherapy itself may increase reliance on OXPHOS in certain resistant tumors, including models with poor response to immune checkpoint blockade. In this context, IACS-010759 has been reported to suppress OXPHOS-supported survival programs, alleviate immunosuppressive metabolic adaptation, and improve the efficacy of radiotherapy (129). Notably, triple combination strategies integrating OXPHOS inhibition, radiotherapy, and anti-PD-1 therapy have produced particularly strong anti-tumor effects in preclinical models, including systemic immune responses and prolonged survival.

Despite these promising results, OXPHOS inhibition is associated with substantial translational challenges. Because mitochondrial respiration is indispensable in many normal tissues, complex I inhibitors can cause significant systemic toxicity, including lactic acidosis and broader metabolic complications (128). Indeed, both phenformin and IACS-010759 have encountered toxicity-related limitations in clinical development. For this reason, the future of this strategy will depend not only on anti-tumor efficacy but also on improved patient selection, optimized dosing schedules, and possibly the development of next-generation agents with greater tumor selectivity. Thus, while OXPHOS inhibition remains a biologically compelling route to radiosensitization, its clinical implementation will require careful balancing of efficacy and tolerability.

### 5.4 Indirect targeting of lactylation machinery: p300/CBP inhibition and delactylation-related strategies

Since lactylation has emerged as a potential mediator of therapeutic resistance, modulation of lactylation-associated machinery has become an area of growing interest. At present, however, this should be framed as indirect targeting rather than as direct inhibition of a dedicated lactyltransferase. Small-molecule inhibitors such as A-485 and C646 suppress the catalytic activity of p300/CBP and thereby reduce histone acetylation and, potentially, histone lactylation when intracellular lactyl-CoA pools favor this reaction (13). In tumor models, p300 inhibition has shown anti-tumor effects and, in some settings, synergy with other targeted therapies (77, 78). These observations raise the possibility that p300 inhibition could enhance radiosensitivity by attenuating acylation-dependent transcriptional programs, but the effect cannot be attributed exclusively to lactylation (130).

This strategy should therefore be interpreted cautiously. p300/CBP are multifunctional chromatin regulators with broad effects on acetylation, transcription, enhancer activity, and cell-state control. Their contribution to lactylation is likely influenced by the intracellular lactyl-CoA/acetyl-CoA ratio and other metabolic acyl pools, rather than by a uniquely lactylation-specific enzyme-substrate relationship (76, 130). Consequently, the biological effects of p300 inhibitors cannot be attributed exclusively to reduced lactylation (78). Nevertheless, in tumors characterized by elevated lactate flux and apparent activation of lactylation-associated transcriptional programs, p300 inhibition remains a plausible indirect approach to suppress lactylation-linked resistance phenotypes.

An alternative concept is to promote the removal of lactylation marks by enhancing delactylase activity. HDAC1–3 and SIRT1–3 have been implicated as lactylation erasers, suggesting that increased activity of these enzymes could, in principle, reduce global or substrate-specific lactylation (81). In experimental settings, overexpression or preserved activity of HDAC3 has been associated with decreased lactylation of proteins such as NBS1 and with impairment of DNA repair-related functions (131). Similarly, SIRT family members may act on selected lactylated substrates, thereby modulating resistance pathways (132). At present, however, there are no well-established pharmacologic activators developed specifically for delactylation enhancement, and most clinically relevant compounds targeting HDACs are inhibitors rather than activators (81). As a result, anti-lactylation therapy remains conceptually attractive but technically underdeveloped. Future progress will require a more precise definition of lactylation enzymes, substrate selectivity, and the relative importance of histone versus non-histone lactylation in radioresistance.

### 5.5 Nanoplatforms and combinatorial metabolic interventions

Nanomedicine offers a potentially useful platform for tumor-selective metabolic intervention, especially in contexts where systemic toxicity limits conventional small-molecule approaches (133). By delivering metabolic enzymes, inhibitors, or responsive payloads directly to tumors, nanoplatforms may improve local efficacy while reducing off-target effects. In the context of lactate targeting, multiple groups have designed systems intended either to deplete intratumoral lactate or to exploit lactate-rich conditions to generate cytotoxic stress (134).

One illustrative strategy is the use of lactate oxidase (LOX)-based nanoplatforms that consume lactate within the tumor microenvironment. In some designs, LOX is combined with catalase or other catalytic components to form nanoreactors that convert lactate into pyruvate and hydrogen peroxide, thereby modulating oxidative stress and local metabolism (135). These systems have been reported to reduce intratumoral lactate levels, inhibit tumor growth, and remodel the immune microenvironment in preclinical models. In melanoma models, for example, lactate-depleting nanoplatforms have been associated with increased cytotoxic T-cell infiltration and improved responses to anti-PD-1 therapy and radiotherapy (136). Other approaches have focused on encapsulating LDH or MCT inhibitors within pH-responsive or tumor-activated nanoparticles to enhance local drug release within acidic tumor tissues (137). Additional multifunctional designs combine metabolic disruption with radiosensitizers, photothermal agents, or immune modulators to achieve multi-pronged therapeutic effects (138).

These strategies are appealing because they may solve several problems simultaneously: selective tumor targeting, local lactate depletion, immune reprogramming, and synergy with radiotherapy (7). However, most lactate-related nanomedicine studies remain at the proof-of-concept stage, and clinical translation faces familiar barriers, including manufacturing complexity, reproducibility challenges, uncertainty in biodistribution, and regulatory burden (139). Thus, while nanoplatforms may eventually help realize the therapeutic potential of lactate-targeted radiosensitization, their current role remains largely preclinical.

### 5.6 Clinical progress and unresolved challenges

Therapeutic strategies targeting lactate metabolism and lactylation are gradually moving toward clinical translation, although progress remains uneven across different classes of intervention. The most advanced efforts to date involve transport and metabolic inhibitors rather than direct anti-lactylation therapies (30). Early clinical experience with AZD3965 has shown that MCT1 inhibition is feasible, but it has also highlighted metabolic toxicity risks, including reversible retinal changes and lactate accumulation (30, 116). LDH-targeting agents have also entered early clinical exploration in selected settings, although their integration with radiotherapy remains underdeveloped, and robust efficacy data remain limited (40, 140). In contrast, interventions aimed specifically at lactylation-related machinery remain largely preclinical and should currently be regarded as indirect, mechanism-guided strategies.

Several overarching challenges need to be addressed before these strategies can be widely implemented. First, metabolic therapies may lack tumor specificity and therefore risk damaging normal tissues, particularly in physiologically stressed patients or in those with metabolic comorbidities. Agents targeting OXPHOS or LDH can perturb systemic metabolism and may increase the likelihood of lactic acidosis or other adverse events (50). Second, intratumoral metabolic heterogeneity means that not all tumors will respond equally to the same metabolic intervention. For example, sensitivity to MCT1 inhibition may depend on low MCT4 expression, whereas the efficacy of LDHA inhibition may be influenced by LDHB expression or by alternative metabolic compensation pathways (29, 40). This underscores the need for biomarker-guided patient stratification, ideally based on molecular profiling, imaging, or other measures of lactate dependency.

Third, the optimal integration of lactate-targeted therapies with radiotherapy, immunotherapy, or DNA damage-targeting drugs remains unresolved (141). The timing and sequence of combination therapy may be particularly important, since excessive metabolic suppression could potentially impair not only tumor cells but also anti-tumor immune cells (142). Likewise, the extent to which lactate-targeted interventions can be used to induce repair vulnerabilities that sensitize tumors to PARP inhibitors or other DNA repair-directed agents remains to be systematically tested (47). Overall, the translational promise of targeting the lactate–lactylation axis is substantial, but meaningful clinical impact will require a more precise understanding of therapeutic windows, resistance mechanisms, and patient selection.

## 6 Outstanding questions and future directions

Despite growing evidence that the lactate–lactylation axis contributes to tumor radioresistance, several major questions remain unresolved (**Table 2**). A central issue is that the enzymatic machinery of lactylation is still incompletely defined. Although p300/CBP, HDAC1–3, and SIRT1–3 have been implicated in the installation or removal of lactylation marks, it remains unclear whether additional substrate-selective writers and erasers exist, whether histone and non-histone substrates are controlled by distinct enzymatic systems, and how enzyme specificity is shaped by metabolic context, protein conformation, or sequence features (76, 143). More broadly, the structural principles governing lactylation remain poorly understood. Which substrates are preferentially modified, under what metabolic conditions lactylation becomes functionally relevant, and how these rules differ from those governing other lysine acylations are all questions that require more systematic investigation. Resolving these issues will be essential not only for mechanistic clarity, but also for the development of inhibitors or modulators with sufficient specificity for translational application.

**Table 2:** Evidence hierarchy and validation priorities for lactate-lactylation mechanisms in tumor radioresistance.

Mechanistic layer	Evidence status	Current interpretation	Validation priority
Lactate metabolism, redox buffering, and DDR support	Established/emerging; strongest support from irradiated tumor models using LDH or MCT perturbation.	Lactate-associated metabolism can support repair capacity and survival, but should be framed as metabolic flexibility rather than a single ATP-only pathway.	Time-resolved lactate tracing, ATP/redox measurements, and rescue experiments after irradiation.
MCT transport and lactate/proton gradients	Established transport biophysics; cancer RT evidence is context dependent.	MCT1 and MCT4 are bidirectional transporters; uptake or export reflects gradients, affinity, capacity, and cell state.	Measure lactate and pH separately; stratify models by MCT1/MCT4 expression and hypoxic status.
Histone lactylation and DDR-related transcription	Emerging; supported by chromatin and cancer stress models, with incomplete direct RT validation.	May create DDR-permissive transcriptional programs, but causality and tumor-type generalizability remain unresolved.	ChIP-seq/CUT&Tag for Kla marks after RT, matched transcriptomics, and locus-specific perturbation.
Non-histone lactylation of repair proteins	Emerging/direct for selected substrates such as NBS1; broader DDR protein evidence remains sparse.	Site-specific lactylation may alter charge, DNA affinity, complex assembly, or damage-site recruitment.	Map modified lysines, test site mutants, and quantify DNA-binding or repair-complex assembly after RT.
Immune microenvironment remodeling	Established/emerging; direct evidence for lactate-rich or acidic niches suppressing antitumor immunity.	Lactate-anion signaling and proton-driven acidosis can jointly impair effector cells, DC function, and macrophage polarization.	Separate low-pH and lactate effects; validate with immune-competent RT models and spatial immune-metabolic profiling.
RNA processing, METTL3, m6A, and non-coding RNAs	Hypothesis-generating in RT; evidence is largely indirect or derived from immune/cancer models without irradiation.	May represent a downstream gene-regulatory layer, but causal linkage to radioresistance is not yet established.	Assess METTL3 conformational or RNA-binding changes, m6A-seq after RT, and functional rescue in irradiated tumors.

A second major challenge lies in defining the spatiotemporal relationship between lactate metabolism, lactylation dynamics, and DNA damage repair. Much of the current evidence is derived from static endpoint analyses, which makes it difficult to distinguish correlation from causation (66, 87). In particular, it remains uncertain whether lactate accumulation precedes repair activation, emerges as a consequence of stress adaptation, or co-evolves dynamically with specific repair pathways after irradiation. Addressing this problem will require more refined approaches capable of monitoring lactate flux, lactylation states, and DNA damage responses in parallel within living systems. Emerging tools such as (13)C-based metabolic tracing, hyperpolarized magnetic resonance spectroscopy, and time-resolved imaging of DNA damage markers may prove especially valuable in defining the sequence of events that links metabolic adaptation to repair activation in irradiated tumors (46, 144). At the same time, future studies will need to move beyond single-layer analyses and adopt integrated multi-omics strategies. Joint profiling of metabolomics, lactyl-proteomics, transcriptomics, chromatin accessibility, and immune-cell states within the same samples could provide a more systems-level view of how the lactate axis supports radioresistance. Particularly promising are spatially resolved approaches that can map lactate-rich niches alongside immunosuppressive cell populations, local chromatin states, and DNA repair signaling *in situ*. Such frameworks may not only improve mechanistic understanding, but also reveal previously unrecognized vulnerability nodes for combination therapy (23).

Equally important is the question of clinical translation. Given the marked heterogeneity of tumor metabolism, reliable biomarkers will be necessary to identify patients most likely to benefit from lactate-targeted radiosensitization. Although FDG-PET provides an indirect measure of glycolytic activity, more direct methods for assessing intratumoral lactate dependence, including lactate-sensitive imaging approaches and magnetic resonance-based metabolic readouts, may ultimately prove more informative. In parallel, tissue- and blood-based biomarkers such as LDHA, MCT1, MCT4, and lactylation-associated marks including H3K18la may help define tumors with high lactate flux or elevated lactylation activity, but these candidates still require rigorous validation in large, clinically annotated cohorts (28). Finally, future work must clarify how lactate-axis targeting can be integrated with other treatment modalities. In immunotherapy, lactate depletion may help convert metabolically suppressed “cold” tumors into more permissive immune environments, providing a rationale for combining radiotherapy, lactate-targeted agents, and immune checkpoint blockade. In the context of DNA damage-directed therapy, LDH inhibition may induce repair vulnerabilities resembling homologous recombination deficiency and thereby increase sensitivity to PARP inhibition (145). These possibilities are attractive, but they remain incompletely tested. Overall, the next phase of the field should move beyond descriptive association toward mechanism-based intervention, biomarker-guided patient selection, and rational combination design, with particular attention to treatment sequence, therapeutic windows, and resistance mechanisms.

## 7 Conclusion

Lactate and lactylation are increasingly recognized as important contributors to tumor radioresistance, but their mechanisms differ in evidential maturity. Established and directly supported mechanisms include lactate-associated metabolic adaptation, redox buffering, DNA damage repair support, and immune suppression in selected irradiated tumor models. Emerging mechanisms include histone and non-histone lactylation-dependent regulation of DDR and immune programs. By contrast, RNA-processing-related mechanisms, including m6A-dependent transcript stability and non-coding RNA-associated regulatory circuits, remain promising but incompletely causal and should be treated primarily as future research directions.

Although preclinical studies have demonstrated the radiosensitizing potential of targeting lactate metabolism and lactylation, substantial barriers still limit clinical translation, including insufficient target specificity, metabolic compensation, tumor heterogeneity, and the complexity of immune regulation. The direct contribution of RNA-level regulation to lactate-driven radioresistance remains insufficiently defined and requires cautious interpretation. Over the next several years, progress in this field will require rigorous mechanistic validation and rationally designed combination strategies. In particular, integrated multi-omics approaches, structural analyses, and spatiotemporal studies will be needed to define lactyl-CoA-dependent lactylation pathways, clarify the dynamic coupling between lactate metabolism and DNA damage responses, and identify predictive biomarkers and tractable therapeutic targets. Ultimately, these efforts may enable more precise and individualized strategies to target the lactate-lactylation axis and overcome radioresistance.
